# Stratified and Quantified Score Construction of Low‐Grade Glioma Samples Based on TP53 Signaling Pathway

**DOI:** 10.1155/bmri/2147185

**Published:** 2026-03-31

**Authors:** Xinlong Ma, Kun Tian, Yongzhang Li, Lipeng Qin, Xin Guo

**Affiliations:** ^1^ Department of Neurosurgery, Yuquan Hospital, Tsinghua University, Beijing, China, tsinghua.edu.cn; ^2^ Department of Breast Surgery, Beijing Haidian Maternal and Child Health Hospital, Beijing, China; ^3^ Research Center, Hebei Province Hospital of Chinese Medicine, Affiliated Hospital of Hebei University of Traditional Chinese Medicine, Shijiazhuang, Hebei, China, hbtcm.edu.cn; ^4^ Hebei Key Laboratory of Turbidity Toxin Syndrome, Shijiazhuang, Hebei, China; ^5^ Department of Neurosurgery, Hebei Province Hospital of Chinese Medicine, Affiliated Hospital of Hebei University of Traditional Chinese Medicine, Shijiazhuang, Hebei, China, hbtcm.edu.cn; ^6^ Department of Pathology and Laboratory Medicine, Department of Pathology, Kanazawa Medical University, Uchinada, Ishikawa, Japan, kanazawa-med.ac.jp

**Keywords:** chemotherapy, heterogeneity, low-grade glioma, TP53

## Abstract

**Background:**

This study is aimed at distinguishing the phenotypes of low‐grade gliomas based on the P53 signaling pathway gene set, revealing the transcriptomic changes in different phenotypes, screening phenotype‐related feature genes, constructing a TP53 score, quantitatively describing TP53‐related phenotypes, and predicting the response of glioma patients to chemotherapy.

**Methods:**

We obtained transcriptomic sequencing data of low‐grade glioma samples from the Cancer Genome Atlas (TCGA) database. Based on the expression levels of genes related to prognosis in the P53 signaling pathway, we performed consensus clustering on glioma samples to describe sample heterogeneity. Through differentially expressed genes (DEGs), we showed the transcriptomic changes between different TP53 phenotypes. We used univariate COX analysis to remove redundant information and retain prognostic factors. We used principal component analysis to retain the first principal component of the prognostic factors as the TP53 score. We elucidated the correlation between the TP53 score and mRNAsi, immune cell subtypes, and EMT markers.

**Results:**

We obtained the TP53 genes and screened 14 prognostic risk factors through univariate COX analysis. Based on prognostic risk factors, we classified glioma samples into TP53 clusters using consensus clustering, describing sample heterogeneity. The prognosis and immune cell microenvironment characteristics differed between TP53 clusters. Among the DEGs between TP53 clusters, we screened 38 as prognostic factors. We used the first principal component of these 38 genes as the TP53 score. The TP53 score was positively correlated with immune cell subtypes and EMT markers and negatively correlated with mRNAsi. High TP53 glioma samples were more sensitive to vorinostat, elesclomol, gefitinib, AICAR, axitinib, and bosutinib.

**Conclusion:**

The TP53 score based on the P53 signaling pathway can describe the heterogeneity of glioma samples and distinguish different immune microenvironment characteristics and prognostic features. A high TP53 score indicates more active epithelial–mesenchymal transition and lower tumor stemness.

## 1. Introduction

Gliomas are the most common primary intracranial tumors, characterized by high recurrence and mortality rates [1–4]. Surgical resection combined with radiotherapy and chemotherapy is the globally recognized principle for glioma treatment [5–7]. The primary goal of glioma treatment is to achieve the maximum safe resection to improve survival rates [8]. However, due to the infiltrative growth of tumors, the location of tumor growth must be considered to minimize damage to the integrity of the nervous system [9, 10]. This poses many limitations for surgical treatment. Surgical resection alone can only moderately extend the median survival of glioma patients. Even with standard treatments such as surgery, radiotherapy, and chemotherapy, the 5‐year survival rate for glioma patients remains low [11]. During treatment, glioma patients face the risk of disease progression and recurrence.

The tumor suppressor factor TP53 is a core factor in maintaining genomic stability and preventing tumorigenesis, with tumor‐suppressing functions such as promoting DNA repair, cell cycle arrest, cell apoptosis, senescence or autophagy, and immune microenvironment regulation [12–16]. Normally, TP53 is maintained at low levels within cells. However, as part of the cellular stress response, TP53 is induced by various stress signals, including DNA damage, oncogene activation, telomere shortening, hypoxia, and nutrient deprivation [17–20]. TP53 may play a pivotal role in the pathogenesis of malignant tumors. Starting from the P53 signaling pathway, we can better describe tumor heterogeneity. Therefore, this study is aimed at distinguishing glioma phenotypes based on the P53 signaling pathway gene set, revealing the transcriptomic changes in different phenotypes, screening phenotype‐related feature genes, constructing a TP53 score, quantitatively describing TP53‐related phenotypes, and predicting the response of glioma patients to chemotherapy.

## 2. Materials and Methods

### 2.1. Data Download

We obtained transcriptomic sequencing data of low‐grade glioma (LGG) samples from the Cancer Genome Atlas (TCGA) database. We only included primary tumor samples, excluding recurrent tumor samples, totaling 511 cases. We integrated the RNA expression data for each sample, establishing an RNA expression matrix for glioma samples, and obtained the corresponding gene symbols from the GFF3 file. To minimize differences in gene expression levels, we performed a logarithmic transformation on the matrix. We also obtained clinical information for glioma samples from TCGA database, including pathological grading, survival time, and survival status.

### 2.2. Single‐Sample Gene Set Enrichment Analysis (ssGSEA)

ssGSEA is an extension of the GSEA method aimed at addressing the challenge of analyzing single samples. The ssGSEA algorithm involves several steps. First, the gene expression values for a given sample are rank‐normalized. Then, the empirical cumulative distribution function is used to calculate the enrichment score (ES) for each gene set. The ES measures the degree of coordinated upregulation or downregulation of a group of genes in the sample. Based on 29 immune‐related gene sets, we used ssGSEA to calculate the ES for each gene set in the collection. The resulting ES can be used to identify the relative infiltration levels of immune cell subtypes in glioma samples.

### 2.3. Differentially Expressed Genes (DEGs)

By screening DEGs, we showed the transcriptomic changes between different immune phenotypes. We used the R language and the DESeq2 package to screen DEGs. DEGs needed to meet the following two criteria simultaneously: FDR < 0.05 and fold change greater than 2.

### 2.4. Consensus Clustering

Ward′s method was used for consensus clustering of glioma samples to describe sample heterogeneity. We sought the *K* value with the flattest cumulative distribution function change within the consensus index range and clustered the samples based on this *K* value.

### 2.5. TP53 Score Construction

First, we determined the DEGs between different immune phenotypes. Then, we used univariate COX analysis to remove redundant information and retain prognostic factors. After determining the ability of prognostic factors to represent immune phenotypes, we used principal component analysis to retain the first principal component of the prognostic factors as the chemokine score.

### 2.6. Enrichment Analysis

Using the human genome as the background, based on the Gene Ontology (GO) and Kyoto Encyclopedia of Genes and Genomes (KEGG) databases, we performed enrichment analysis of DEGs using the R package (clusterProfiler). KEGG pathways and GO terms were retained, with the screening condition *q* < 0.05.

### 2.7. Statistical Analysis

All analyses were performed using R software Version 3.3 and R packages. All statistical tests were two‐sided, with *p* values < 0.05 considered statistically significant.

## 3. Results

### 3.1. Consensus Clustering Based on TP53 Genes

We obtained P53 signaling pathway genes (Table S1) and screened 14 prognostic factors through univariate COX analysis (Table S2). There was extensive correlation among these risk factors (Figure 1a). Through consensus clustering, we stratified LGG samples to describe different sample phenotypes. Referring to the clarity of the consensus matrix heatmap (Figure 1b) and the flatness of the cumulative distribution function within the consensus index range (Figure 1c), we divided the samples into two types: TP53 Cluster A (*n* = 400) and TP53 Cluster B (*n* = 111). We validated the reliability of the consensus clustering results through prognostic information and principal component analysis. TP53 Clusters A and B had different prognostic phenotypes, with TP53 Cluster A having a more favorable prognosis (Figure 1d). The expression levels of P53 signaling pathway genes in TP53 Clusters A and B are shown in Figure 1e. In the two‐dimensional coordinate system formed by the first and second principal components based on P53 signaling pathway genes, TP53 clusters were enriched in different regions, indicating high accuracy and good reliability of the consensus clustering stratification (Figure 1f). PD‐L1 (Figure 1g) and CTLA4 (Figure 1h) were highly expressed in TP53 Cluster B, while the infiltration levels of immune cell subtypes (Figure 1i) and immune score (Figure 1j) showed similar differential trends.

Figure 1Consensus clustering based on prognostic factors derived from the P53 signaling pathway gene set in low‐grade gliomas. (a) Correlation between prognostic factors screened from the P53 signaling pathway gene set. (b) Consensus matrix of consensus clustering based on prognostic factors. (c) Cumulative distribution function of consensus clustering based on prognostic factors. (d) Survival analysis of TP53 clusters. (e) Heatmap of P53 signaling pathway gene set expression between TP53 clusters. (f) Principal component analysis of TP53 clusters based on P53 signaling pathway gene set expression. Differences in (g) PD‐L1 and (h) CTLA‐4 between TP53 clusters. (i) Differences in immune cell subtypes between TP53 clusters. (j) Differences in immune scores between TP53 clusters. Note:  ^∗∗∗^ *p* < 0.001.

**Figure:**
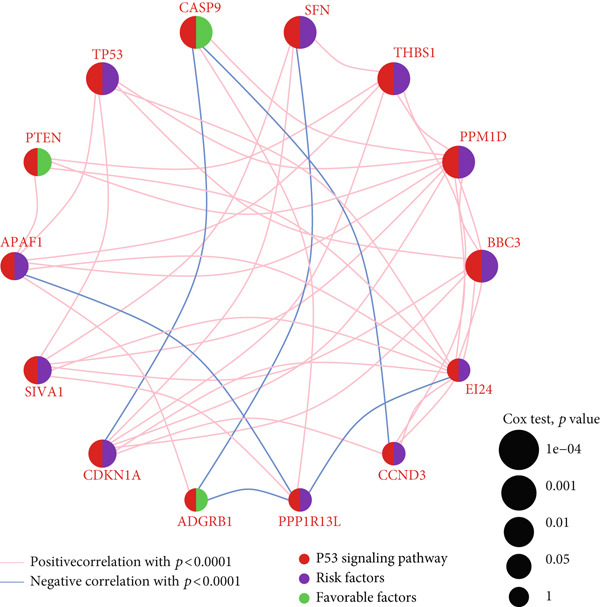
(a)

**Figure:**
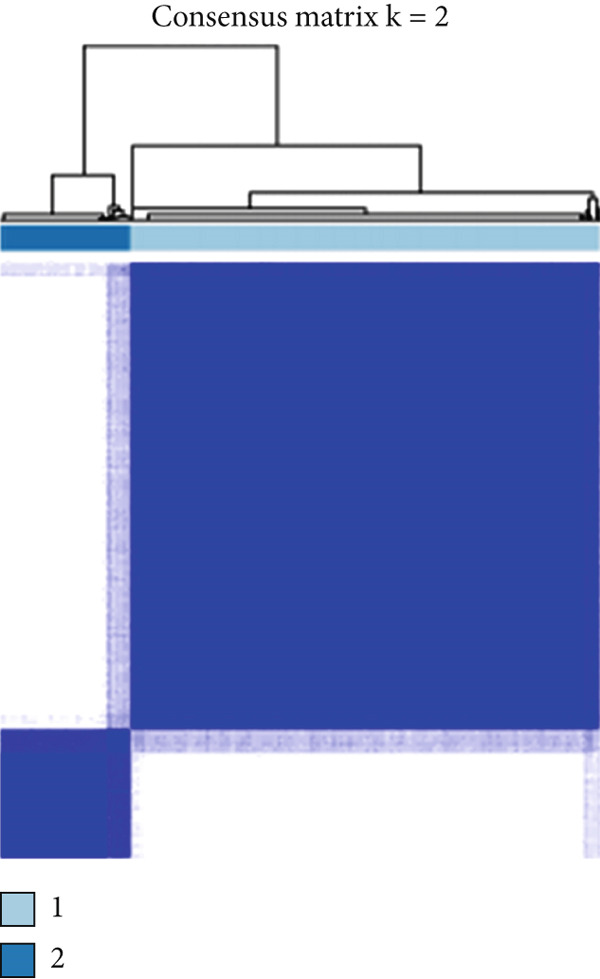
(b)

**Figure:**
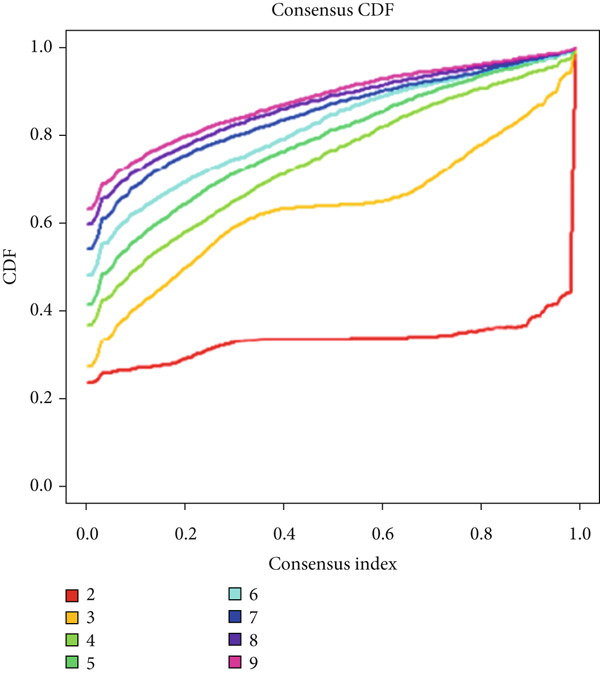
(c)

**Figure:**
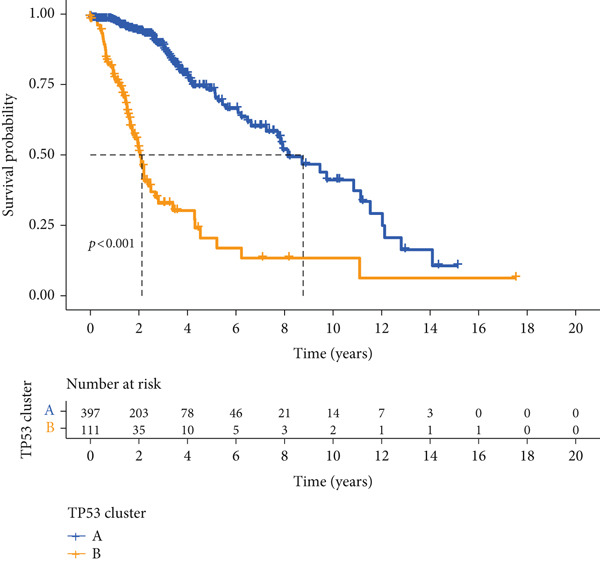
(d)

**Figure:**
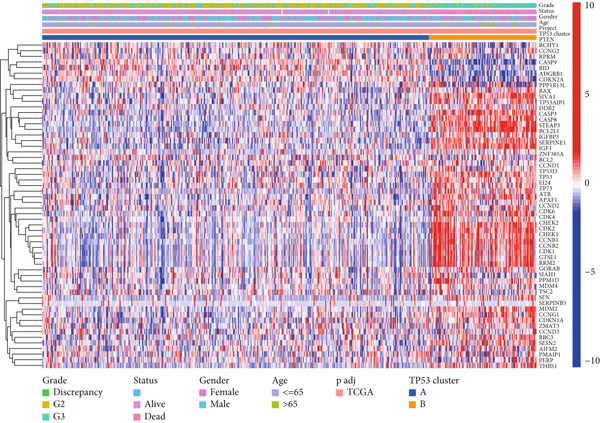
(e)

**Figure:**
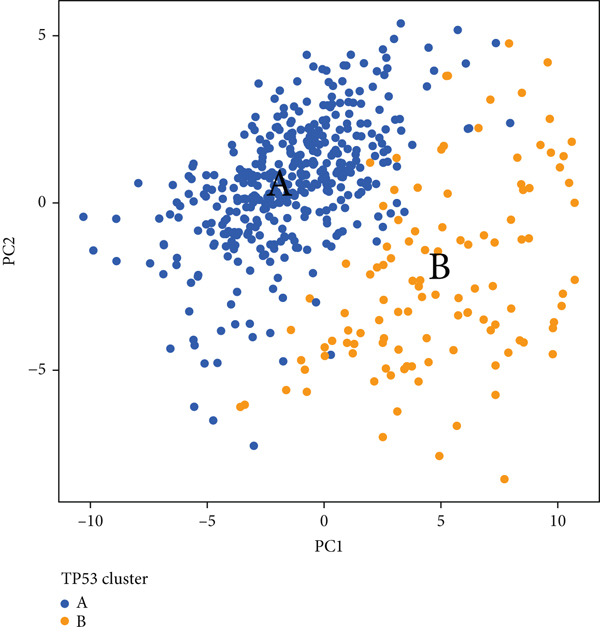
(f)

**Figure:**
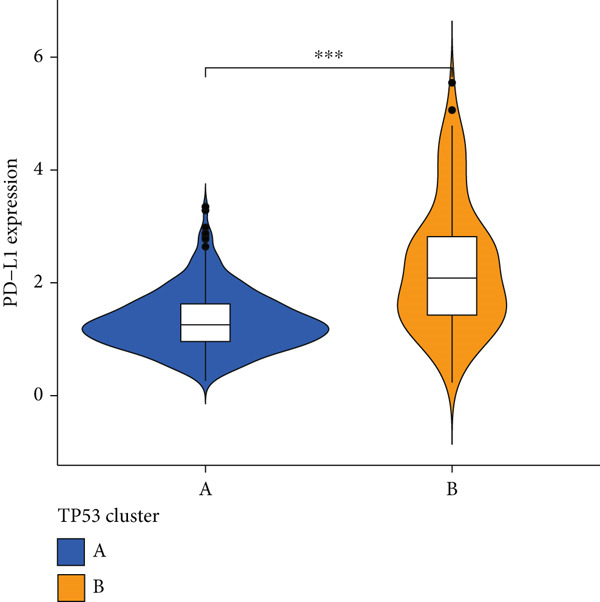
(g)

**Figure:**
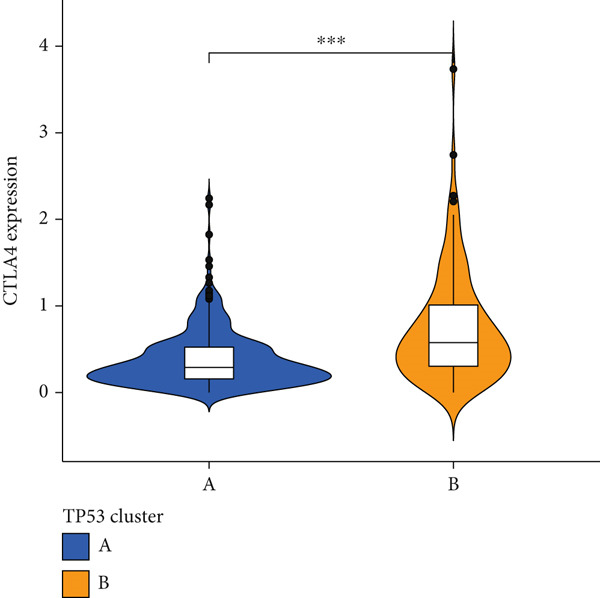
(h)

**Figure:**
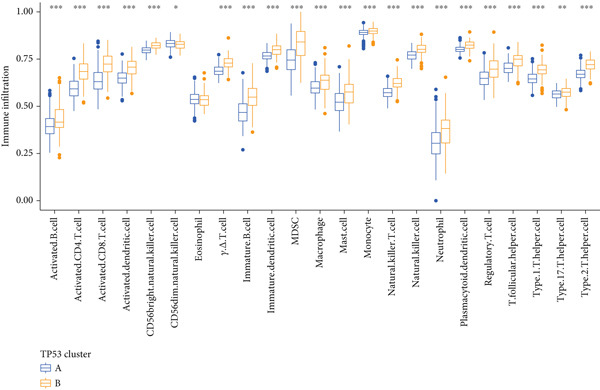
(i)

**Figure:**
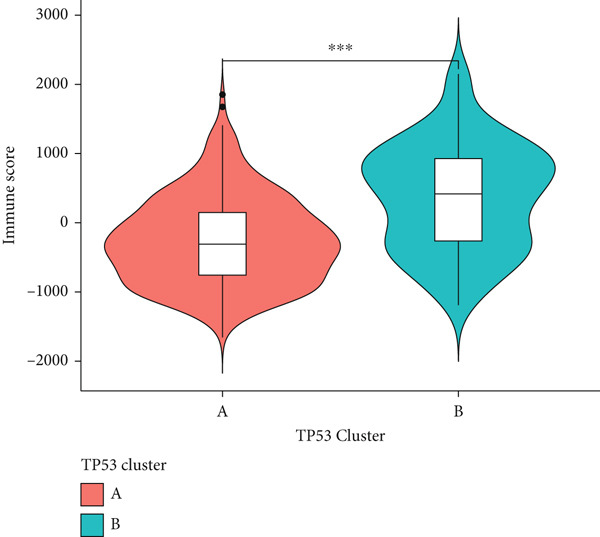
(j)

### 3.2. Transcriptomic Changes Between TP53 Clusters

We confirmed the reliability of consensus clustering through PCA, survival analysis, comparison of P53 signaling pathway gene expression, and immune score comparison, accurately describing the heterogeneity of LGGs. To further explore the transcriptomic changes between different TP53 clusters and reveal their potential differentiation mechanisms, we performed a series of enrichment analyses. GSVA showed that compared to TP53 Cluster A, some pathway gene sets were highly expressed in TP53 Cluster B, including ECM–receptor interaction, glycosaminoglycan degradation, P53 signaling pathway, mismatch repair, JAK‐STAT signaling pathway, and leukocyte transendothelial migration (Figure 2a). We screened DEGs between TP53 clusters. The biological process terms enriched by these DEGs included extracellular matrix organization, extracellular structure organization, and external encapsulating structure organization. The cellular component terms enriched by these DEGs included collagen‐containing extracellular matrix, complex of collagen trimers, and collagen trimer. The molecular function terms enriched by these DEGs included extracellular matrix structural constituent, extracellular matrix structural constituent conferring tensile strength, and platelet‐derived growth factor binding (Figure 2b). The KEGG pathways enriched by these DEGs included ECM–receptor interaction, Th1 and Th2 cell differentiation, and antigen processing and presentation (Figure 2c).

Figure 2Transcriptomic alterations between TP53 clusters. (a) Gene set variation analysis (GSVA) between TP53 Clusters A and B. (b) GO enrichment analysis of differentially expressed genes (DEGs) between TP53 clusters. (c) KEGG pathway enrichment analysis of DEGs between TP53 clusters.

**Figure:**
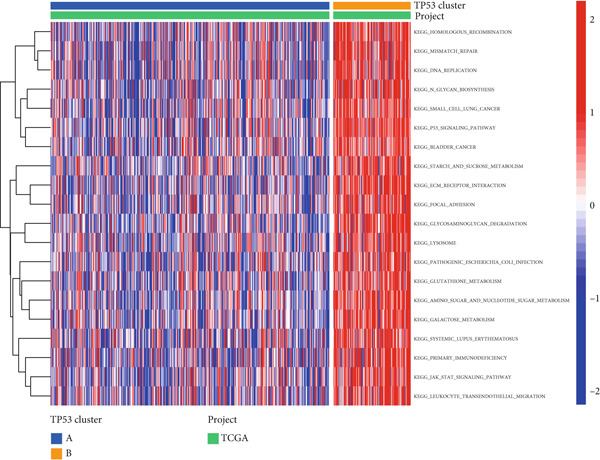
(a)

**Figure:**
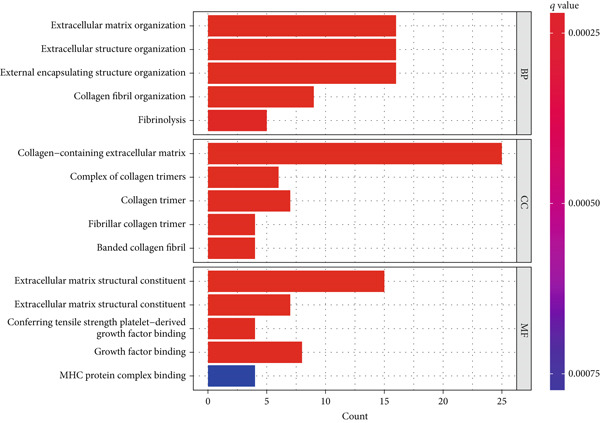
(b)

**Figure:**
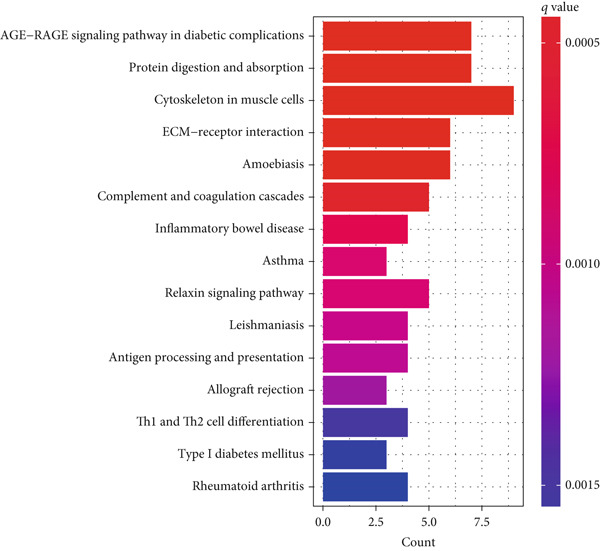
(c)

### 3.3. Consensus Clustering Based on Prognosis‐Related DEGs

Based on the expression of common DEGs between TP53 clusters (Table S3), we performed consensus clustering stratification of LGG samples to verify the ability of DEGs to characterize TP53‐related phenotypes and prepare for the construction of the scoring system. We screened prognosis‐related DEGs through univariate COX analysis, reducing the scale of DEGs and removing redundant information. Considering the clarity of the consensus matrix heatmap (Figure 3a) and the flatness of the cumulative distribution function within the consensus index range (Figure 3b), we divided the LGG samples into two gene clusters. Gene Cluster A was characterized by a good prognosis, while Gene Cluster B was the opposite (Figure 3c). The expression levels of prognosis‐related DEGs in Gene Clusters A and B are shown in Figure 3d. In the P53 signaling pathway gene set, prognostic risk factors were highly expressed in Gene Cluster B, while prognostic protective factors (CASP3, PTEN, and ADGRB1) were upregulated in Gene Cluster A (Figure 3e), consistent with the survival analysis trends. Immune cell subtypes were highly expressed in Gene Cluster B (Figure 3f).

Figure 3Consensus clustering based on prognostically differentially expressed genes (DEGs). (a) Consensus matrix of consensus clustering based on prognostic DEGs. (b) Cumulative distribution function of consensus clustering based on prognostic DEGs. (c) Survival analysis of gene clusters. (d) Heatmap of prognostic DEG expression between gene clusters. (e) Differences in the expression levels of prognostic factors from the P53 signaling pathway gene set between gene clusters. (f) Differences in immune cell subtype infiltration between gene clusters. Note:  ^∗^ *p* < 0.05,  ^∗∗^ *p* < 0.01, and  ^∗∗∗^ *p* < 0.001.

**Figure:**
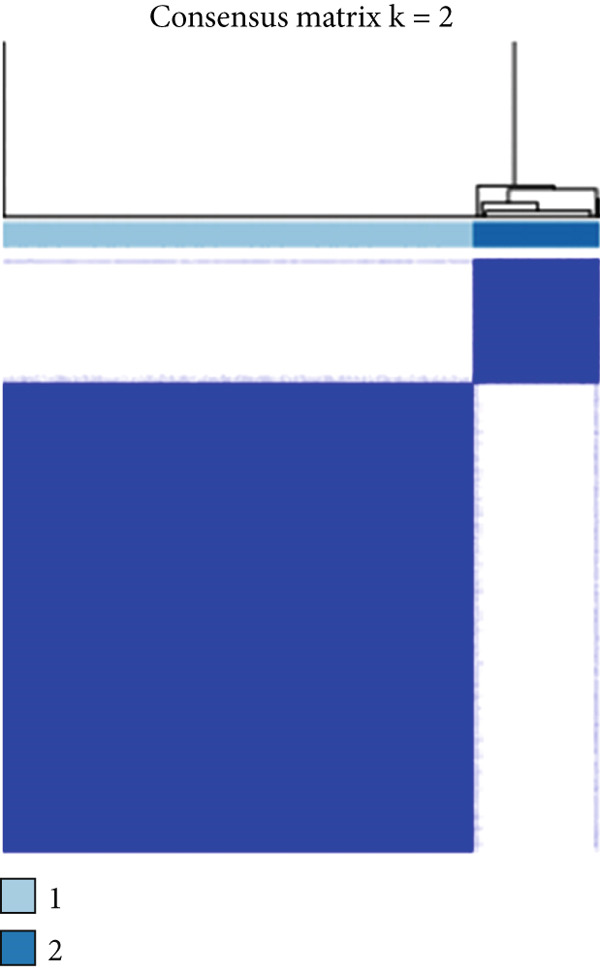
(a)

**Figure:**
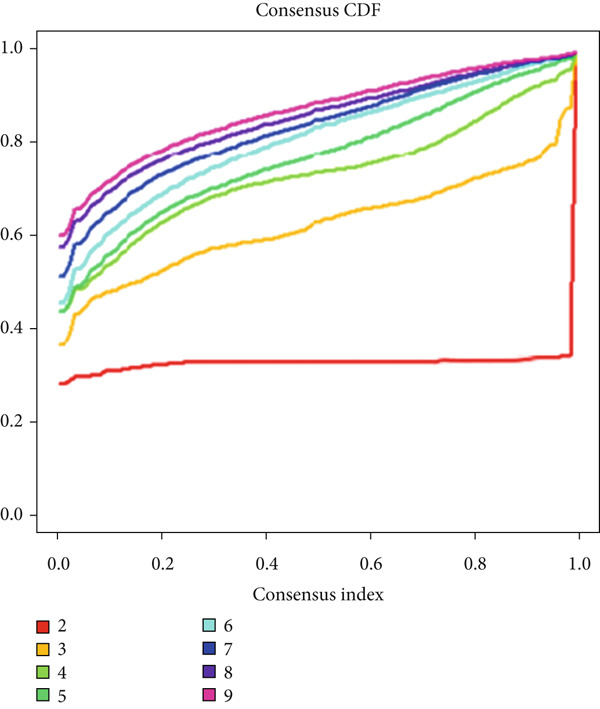
(b)

**Figure:**
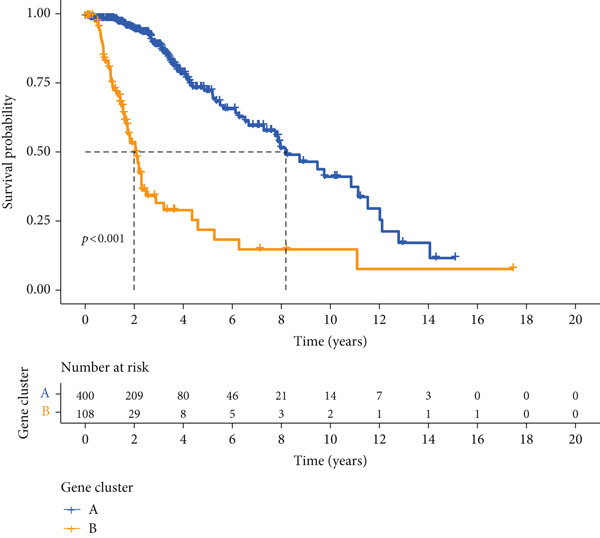
(c)

**Figure:**
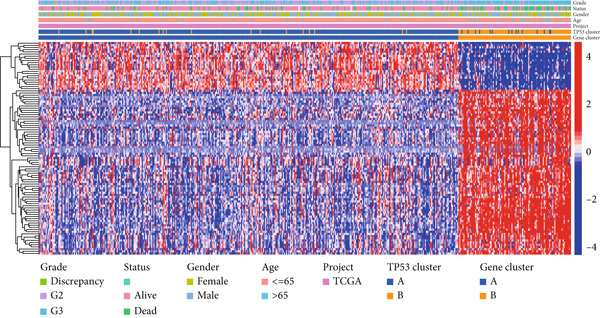
(d)

**Figure:**
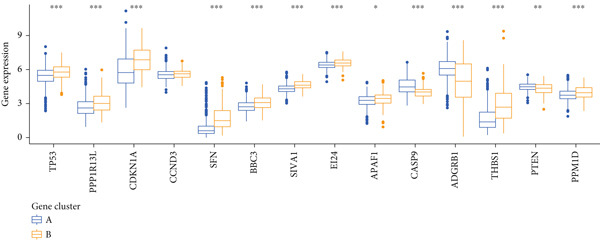
(e)

**Figure:**
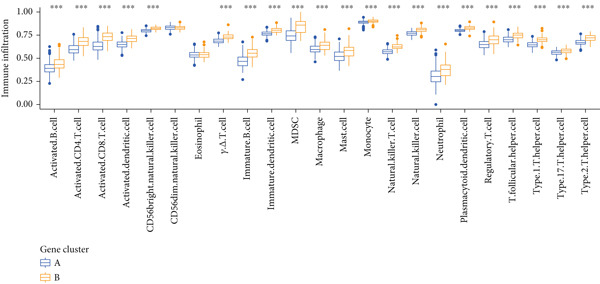
(f)

### 3.4. TP53 Score Construction

To better quantitatively describe the TP53‐related phenotypes of LGG samples, we established a TP53 scoring system, aiming for its more convenient and intuitive application in clinical practice. We used principal component analysis, taking the first principal component of prognosis‐related DEGs as the TP53 score for LGG samples. We selected the most significant cutoff value and divided the LGG samples into high and low TP53 score groups. Patients with low TP53 scores were characterized by a good prognosis (Figure 4a). The TP53 score was higher in Gene Cluster B than in Gene Cluster A (Figure 4b) and higher in TP53 Cluster B than in TP53 Cluster A (Figure 4c). The distribution of LGG samples in TP53 clusters, gene clusters, and TP53 groups, as well as their survival status, is shown in Figure 4d. The TP53 score was positively correlated with the infiltration levels of most immune cell subtypes (Figure 4e) and epithelial–mesenchymal transition (EMT) markers (Figure 4f). To evaluate whether the TP53 score could accurately characterize the stemness of LGGs, we performed an analysis combining mRNAsi and the TP53 score. Patients with high TP53 scores had lower mRNAsi (Figure 4g). The TP53 score was negatively correlated with mRNAsi (*r* = −0.6, *p* < 0.001, Figure 4h). Patients with high mRNAsi gliomas had a better prognosis (Figure 4i). We stratified glioma samples based on mRNAsi and TP53 and compared the prognostic differences between the stratified samples. In patients with high TP53 scores, the prognosis of patients with high mRNAsi and low mRNAsi was difficult to distinguish. In patients with low TP53 scores, patients with high mRNAsi had a better prognosis than those with low mRNAsi (Figure 4j). We further analyzed the relationship between the TP53 score and clinical characteristics. A higher proportion of Grade 3 glioma samples was found in patients with high TP53 scores (Figure 4k). The TP53 score of Grade 3 glioma samples was higher than that of Grade 2 glioma samples (Figure 4l).

Figure 4TP53 score construction. (a) Survival analysis of low and high TP53 groups. (b) Differences in TP53 scores between gene clusters. (c) Differences in TP53 scores between TP53 clusters. (d) Distribution of low‐grade glioma samples in TP53 clusters, gene clusters, and TP53 score groups with different survival statuses. (e) Correlation between TP53 score and immune cell subtype infiltration. (f) Correlation between TP53 score and epithelial–mesenchymal transition markers. (g) Differences in mRNAsi between low and high TP53 clusters. (h) TP53 score is negatively correlated with mRNAsi. (i) Survival analysis of high and low mRNAsi groups. (j) Survival analysis of patients grouped by mRNAsi and TP53 score. (k) Proportion of Grade 2 and Grade 3 patients in high and low TP53 groups. (l) Differences in TP53 scores among different pathological grades of glioma samples. Note:  ^∗^ *p* < 0.05. H, high; L, low.

**Figure:**
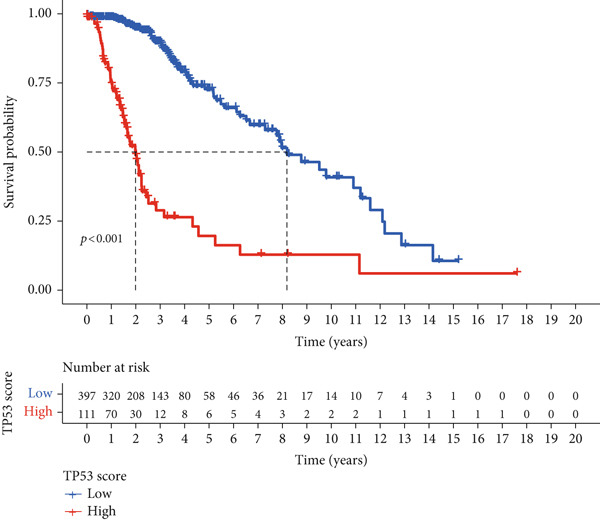
(a)

**Figure:**
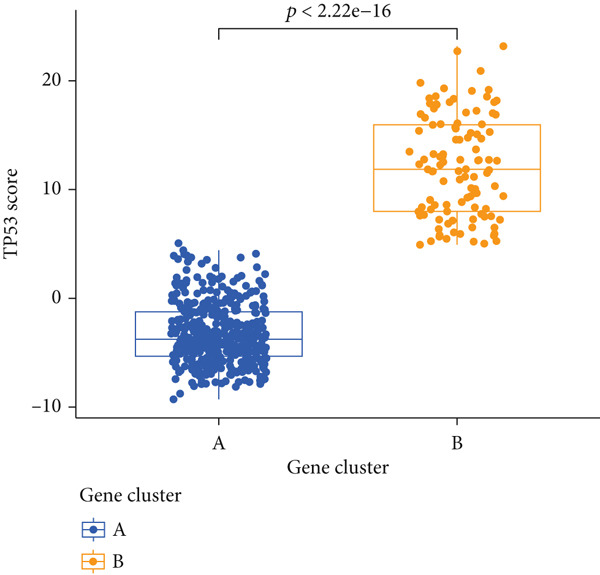
(b)

**Figure:**
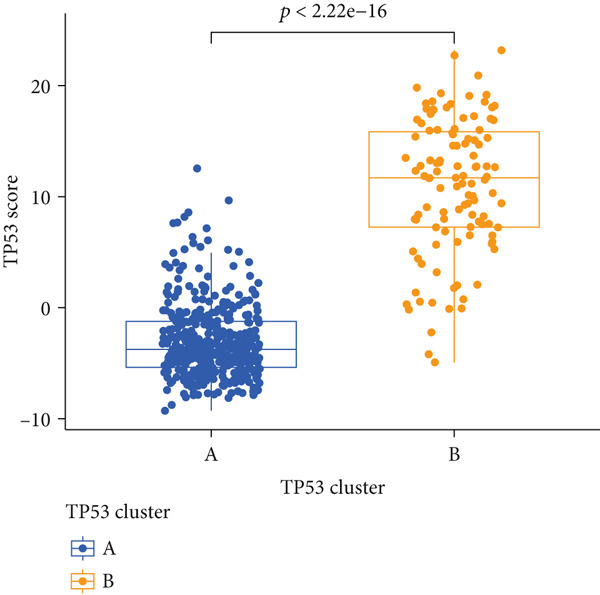
(c)

**Figure:**
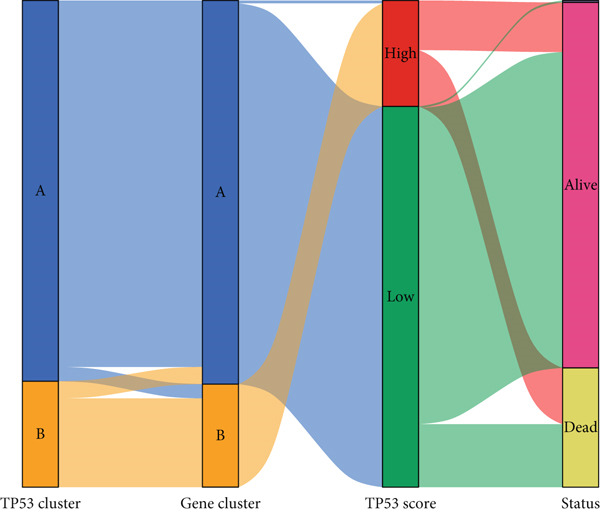
(d)

**Figure:**
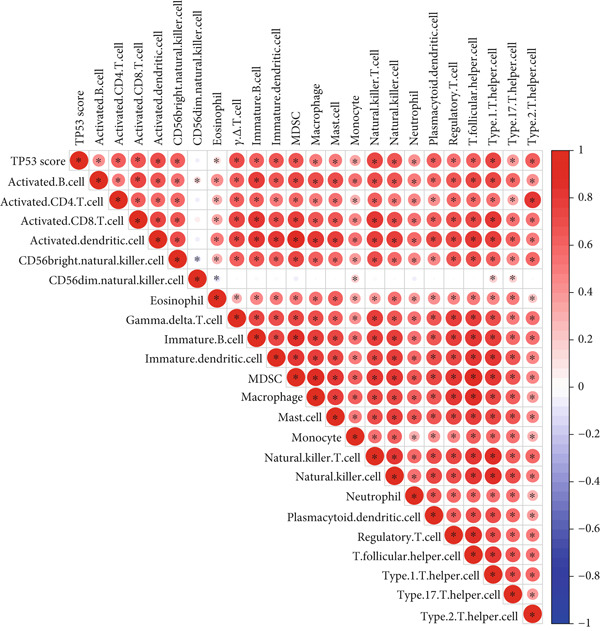
(e)

**Figure:**
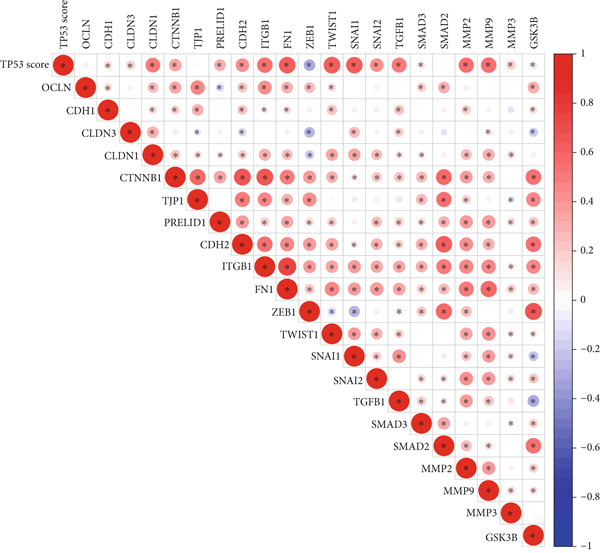
(f)

**Figure:**
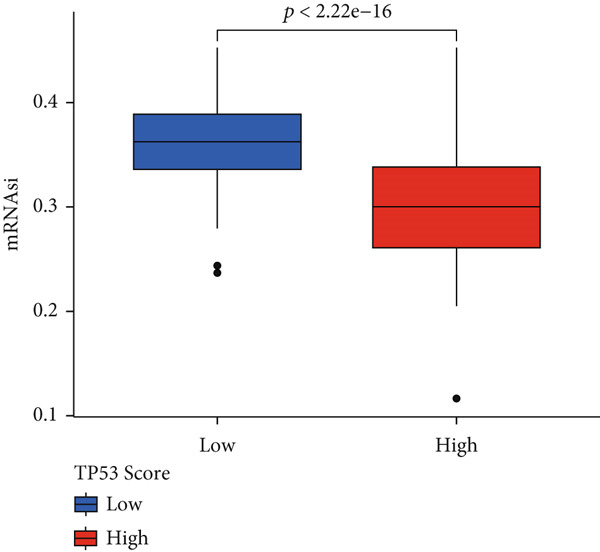
(g)

**Figure:**
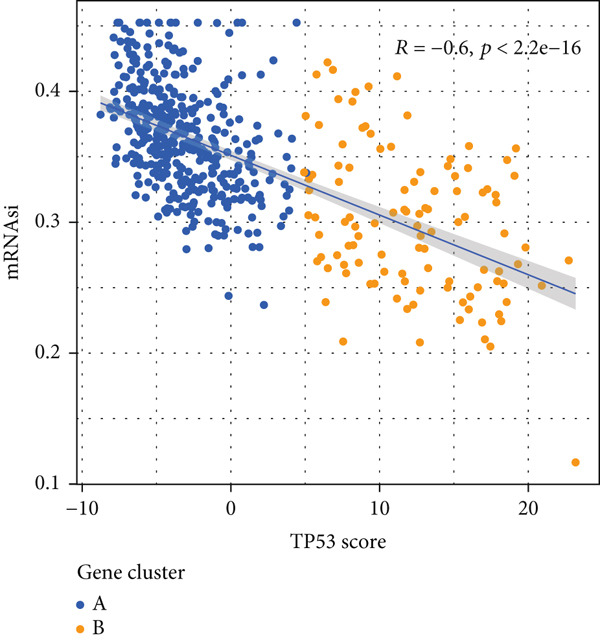
(h)

**Figure:**
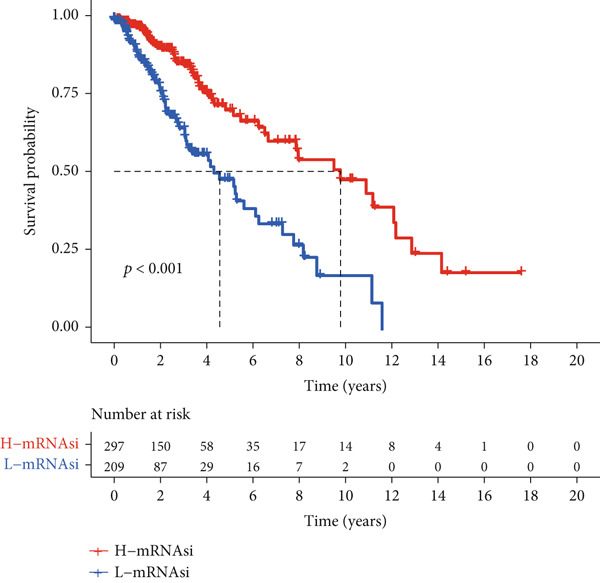
(i)

**Figure:**
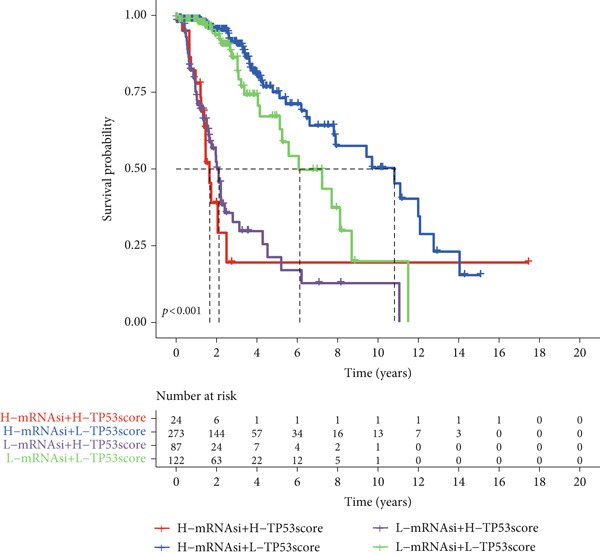
(j)

**Figure:**
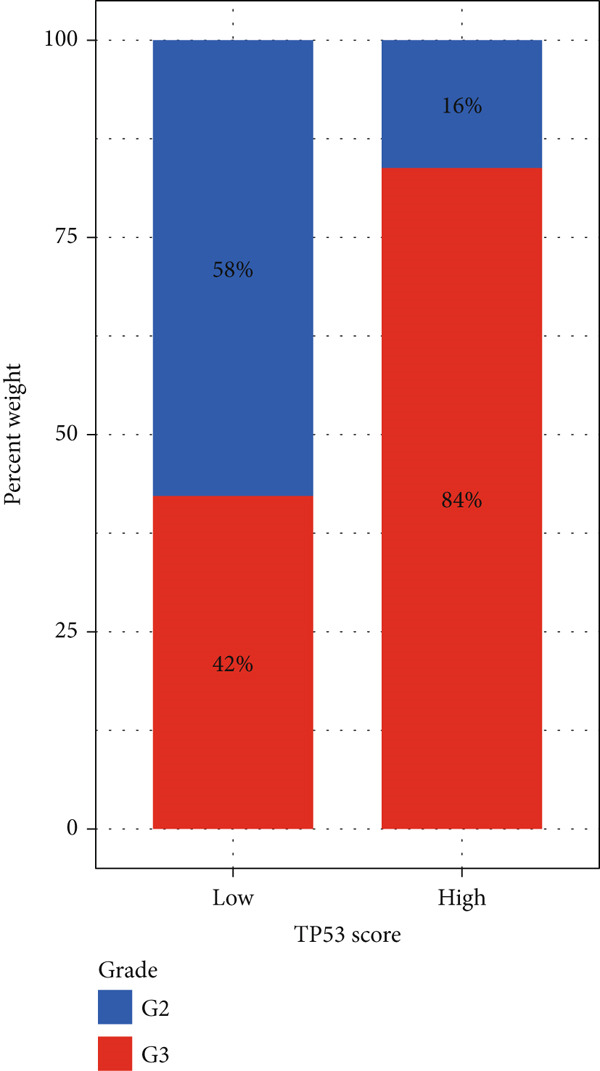
(k)

**Figure:**
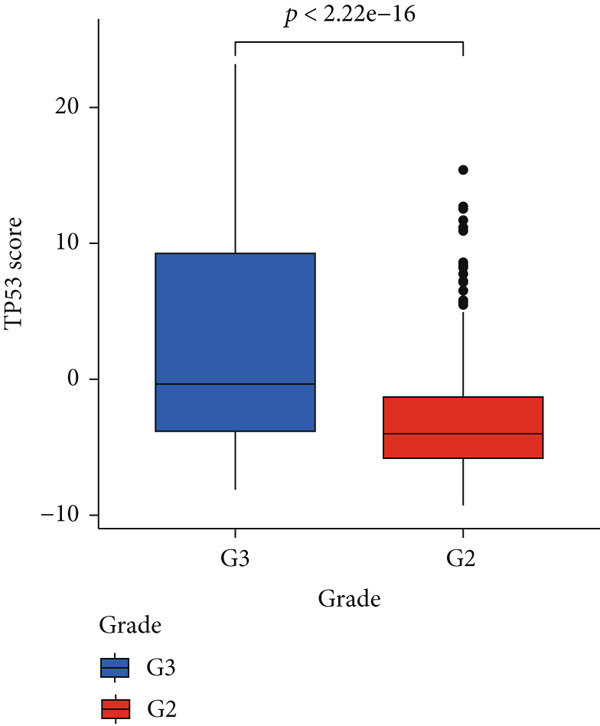
(l)

**Figure 5:**
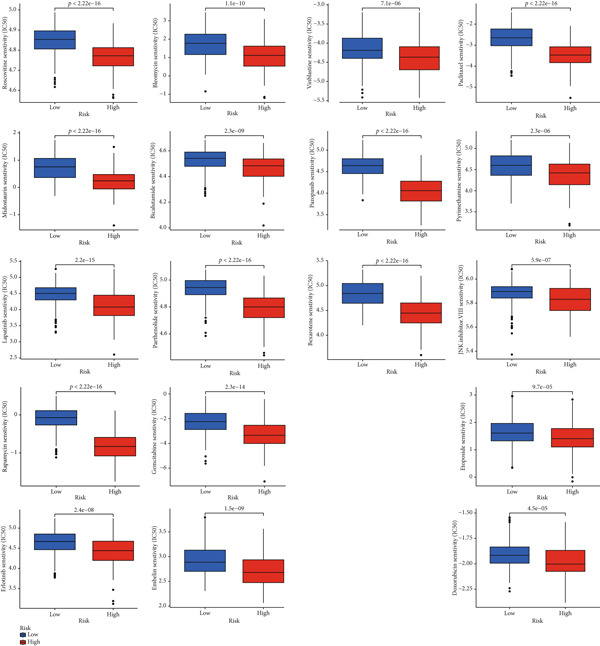
Chemotherapy drug sensitivity analysis based on TP53 score in low‐grade glioma.

**Figure 6:**
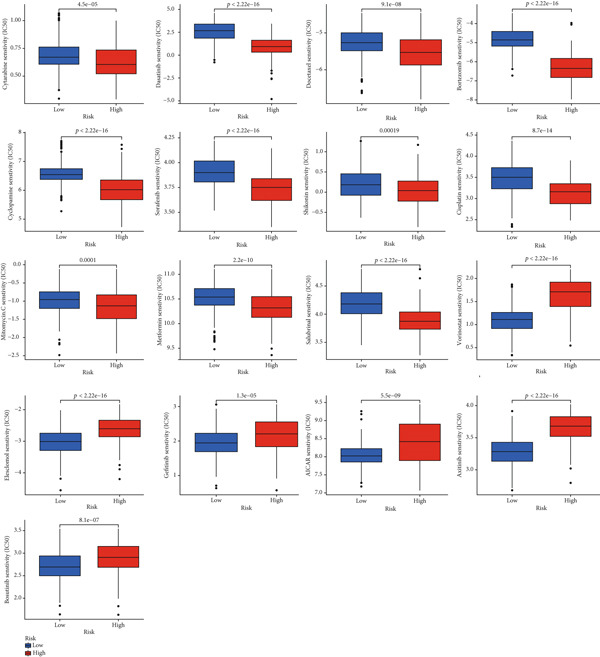
Differential chemotherapy response in TP53 phenotypes of low‐grade glioma.

Glioma samples with high TP53 scores had lower IC50 values for most chemotherapy drugs, indicating that glioma patients with high TP53 scores may be more sensitive to chemotherapy. For these drugs (vorinostat, elesclomol, gefitinib, AICAR, axitinib, and bosutinib), low TP53 glioma samples showed lower IC50 values (Figures 5 and 6).

## 4. Discussion

Authors should discuss the results and how they can be interpreted from the perspective of previous studies and of the working hypotheses. The findings and their implications should be discussed in the broadest context possible. Future research directions may also be highlighted.

TP53 promotes cell cycle arrest and apoptosis by activating caspases to regulate the cell cycle and DNA damage–induced mutations and by inactivating antiapoptotic proteins and activating proapoptotic factors [21–26]. Cell growth is regulated by cyclin‐dependent kinases (CDKs) and cyclins. In the G1 phase, CDK4 and CyclinD can initiate cell division [27]. TP53 can also inhibit the progression of the cell cycle from G1 to S phase by inhibiting the CyclinE‐CDK2 pathway [28–31]. Mild or transient stress can initiate the TP53 response, such as cell cycle arrest and DNA repair [32, 33]. After DNA damage and TP53 activation, the transcription of p21 is induced, causing cell cycle arrest in the G1 phase [33–35]. In addition to cell cycle arrest and apoptosis, TP53 is involved in metabolic regulation. TP53 inhibits glycolysis and promotes oxidative phosphorylation through the nuclear factor kB signaling pathway, blocking the Warburg effect [36–39]. TP53 can negatively regulate glycolysis by downregulating the expression of glucose transporters and reducing intracellular levels of fructose‐2,6‐bisphosphate; at the same time, TP53 can promote mitochondrial oxidative phosphorylation, maintaining mitochondrial integrity; furthermore, TP53 can reduce fatty acid synthesis and control lipid metabolism by activating fatty acid oxidation and is involved in the regulation of reactive oxygen species [40–42]. TP53 promotes the body′s antitumor immune response in multiple ways. Studies have shown that TP53 activation can directly enhance the expression and function of Toll‐like Receptors 3 and 8 on tumor cells, thereby enhancing TLR‐induced cell death and inflammation. The loss or inactivation of TP53 can induce the differentiation of immune cells and their tumor‐promoting effects, including impairing the differentiation of regulatory T cells, increasing the levels of circulating neutrophils that promote tumor growth, and enhancing the ability of basophils to express inflammatory cytokines [43–45]. TP53 is also involved in the regulation of EMT and cellular autophagy [12, 46, 47].

Based on the special role of TP53 in tumorigenesis and progression, we screened prognostic factors for gliomas from the P53 signaling pathway gene set. There was extensive correlation among these prognostic factors. Therefore, we expected to classify samples into different phenotypes based on the expression levels of these prognostic factors to distinguish the phenotypes of glioma samples and describe their heterogeneity. We used consensus clustering to stratify glioma samples. TP53 clusters had different immune cell landscapes and prognostic phenotypes. Type A samples had low immune cell infiltration levels but good prognosis, while TP53 Cluster B was the opposite. Abnormal changes in the P53 signaling pathway can prevent immune cells from migrating toward tumors, promoting immune escape. One study showed that activating the P53 signaling pathway can prevent tumor cells from achieving immune escape and reshape the tumor microenvironment, benefiting cancer patients [48]. TP53 can also enhance the body′s immune surveillance by preventing oxidative stress, inhibiting excessive inflammation, and activating the differentiation of tumor‐suppressive immune cells, making it an important tumor suppressor [49–52]. However, TP53 mutations (mtTP53) have been found in about 50% of tumors and are the most commonly mutated genes in cancer. Studies have shown that mtTP53 can participate in the formation and activation of inflammation, antigen presentation, and activation of natural killer cells and other immune response processes, disrupting the immune homeostasis maintained by TP53 and promoting immune escape and tumorigenesis. Immunotherapy has become a powerful clinical strategy for treating cancer. Based on the role of mtTP53 in immunity, mtTP53‐specific vaccines have been developed, demonstrating the potential of mtTP53 as an immunotherapy antigen target. Additionally, mtTP53 can serve as a predictive biomarker for programmed cell death protein 1/programmed cell death ligand 1 inhibitor therapy, playing an important role in increasing cancer detection rates and PD‐1/PD‐L1 treatment response rates. In gliomas, the enrichment of immune cells within tumors predicts poor patient prognosis. This may be related to the presence of the blood–brain barrier, as high levels of immune cell infiltration may disrupt the originally closed environment. The application of immunotherapy in gliomas has been slow, which may also be related to this. In TP53 Cluster A, the expression levels of PD‐L1 and CTLA4 were lower. We believe this is due to the low infiltration levels of immune cells. In the principal component analysis based on the P53 signaling pathway gene set, TP53 Clusters A and B had good distinguishability. This indicates that our classification is reliable, and the activity of the P53 signaling pathway in different clusters may differ.

To further characterize the transcriptomic landscape between different phenotypes and reveal the mechanisms of phenotypic differentiation, we performed GSVA between every two TP53 clusters. Some gene sets (including ECM–receptor interaction, glycosaminoglycan degradation, P53 signaling pathway, mismatch repair, JAK‐STAT signaling pathway, and leukocyte transendothelial migration) were highly expressed in TP53 Cluster B. This may be the potential mechanism of phenotypic differentiation or the result of TP53‐related phenotypic pathway activation differences. The difference in the ECM–receptor interaction pathway between different clusters may suggest the involvement of the P53 signaling pathway in the regulation of EMT.

TP53 clusters had different immune cell infiltration levels and prognostic phenotypes. To reveal their potential differentiation mechanisms, we explored the transcriptomic changes between TP53 clusters. GO enrichment analysis and KEGG suggested that the functional differences in the transcriptomes of different clusters were mainly reflected in two directions: immune response and EMT. This is consistent with the GSVA results. We screened prognostic factors from DEGs through univariate COX analysis. We attempted to construct a scoring system based on these prognostic factors to better describe TP53‐related phenotypes. We performed consensus clustering on glioma samples again based on prognostic factors. Different gene clusters showed different prognostic and immune characteristics. Moreover, gene clusters also showed different TP53‐related phenotypes. These trends were all consistent with TP53 clusters. Therefore, prognostic factors in DEGs can accurately characterize TP53‐related phenotypes. We constructed the TP53 score using principal component analysis. The TP53 score is based on the P53 signaling pathway and is expected to reflect the heterogeneity of glioma samples. The distribution of glioma samples in TP53 clusters, gene clusters, and TP53 scores was generally consistent. The TP53 score was correlated with immune cell subtypes and EMT markers. This is what we expected and is consistent with previous research suggesting that the P53 signaling pathway is involved in shaping the immune microenvironment and regulating EMT. Additionally, we found that the TP53 score was correlated with mRNAsi. Different pathological grades had different TP53 scores. Based on the TP53 score, we predicted the sensitivity of patients to chemotherapy drugs. Glioma samples with high TP53 scores had lower IC50 values for most chemotherapy drugs, indicating that glioma patients with high TP53 scores may be more sensitive to chemotherapy. For these drugs (vorinostat, elesclomol, gefitinib, AICAR, axitinib, and bosutinib), low TP53 glioma samples showed lower IC50 values.

## 5. Conclusions

The TP53 score based on the P53 signaling pathway can describe the heterogeneity of glioma samples and distinguish different immune microenvironment characteristics and prognostic features. A high TP53 score indicates more active EMT and lower tumor stemness.

**Nomenclature**
DEGs: differentially expressed genes
ssGSEA: single‐sample Gene Set Enrichment Analysis
GO: Gene Ontology
KEGG: Kyoto Encyclopedia of Genes and Genomes
LGG: low‐grade glioma
CDKs: cyclin‐dependent kinases
mtTP53: TP53 mutations

## Conflicts of Interest

The authors declare no conflicts of interest.

## Author Contributions

Conceptualization: X.G. Methodology: X.M., L.Q., and K.T. Software: K.T. Formal analysis: Y.L. Investigation: X.M. Data curation: X.M. Writing—original draft preparation: X.M. Writing—review and editing: X.G. Supervision: X.G. X.M. and K.T. are co‐first authors.

## Funding

This study was funded by the Natural Science Foundation of Hebei Province (10.13039/501100003787) (H2024423028) and the Hebei Yanzhao Golden Platform Talent Gathering Plan Backbone Talent Project (B2024026).

## Supporting information


**Supporting Information** Additional supporting information can be found online in the Supporting Information section. P53 signaling pathway genes screened 14 prognostic factors through univariate COX analysis, and the expression of common DEGs between TP53 clusters was provided in the supporting information tables.

## Data Availability

The data comes from publicly available databases and can be publicly applied. All data generated or analyzed during this study are included in its supporting information files.
