# Supplementary material for: Stratified and Quantified Score Construction of Low‐Grade Glioma Samples Based on TP53 Signaling Pathway
Source: Biomed Res Int. 2026 Mar 31;2026:2147185. doi: 10.1155/bmri/2147185 (PMC13140867; doi:10.1155/bmri/2147185)
Supplement: Supplementary file 1 — Supporting Information Additional supporting information can be found online in the Supporting Information section. P53 signaling pathway genes screened 14 prognostic factors through univariate COX analysis, and the expression of common DEGs between TP53 clusters was provided in the supporting information tables. [file BMRI-2026-2147185-s001.zip › supple table legends 626.docx]

**Table S1.** List of genes analyzed in the study. This table contains the complete set of genes evaluated for their association with the studied phenotype.

**Table S2.** Hazard ratios (HR) and statistical significance of gene associations. The table presents HR values with 95% confidence intervals (HR.95L and HR.95H) and p-values for each gene, indicating their prognostic significance.

**Table S3.** Differentially expressed genes with log2 fold changes (logFC), p-values (P.Value), and false discovery rates (FDR). This table highlights genes with significant expression differences between compared groups.
